# Wnt10b promotes hair follicles growth and dermal papilla cells proliferation via Wnt/β-Catenin signaling pathway in Rex rabbits

**DOI:** 10.1042/BSR20191248

**Published:** 2020-02-04

**Authors:** Zhenyu Wu, Yanli Zhu, Hongli Liu, Gongyan Liu, Fuchang Li

**Affiliations:** 1 College of Animal Science and Technology, Shandong Agricultural University, Tai’an 271018, China; 2 Shandong Provincial Key Laboratory of Animal Biotechnology and Disease Control and Prevention, Shandong Agricultural University, 61 Daizong Street, Taian 271018, China; 3 Shandong Provincial Engineering Technology Research Center of Animal Disease Control and Prevention, Shandong Agricultural University, 61 Daizong Street, Taian 271018, China

**Keywords:** dermal papilla cells, hair follicles, Rex-rabbit, Wnt/β-Catenin Pathway, Wnt10b

## Abstract

Wnt signaling plays an important role in the growth and development of hair follicles (HFs). Among the signaling molecules, Wnt10b was shown to promote the differentiation of primary skin epithelial cells toward the hair shaft and inner root sheath of the HF cells in mice *in vitro*. Whisker HFs were isolated from Rex rabbits and cultured *in vitro* to measure hair shaft growth. Meanwhile, dermal papilla cells (DPCs) were isolated and cultured *in vitro*. Treatment with AdWnt10b or the Wnt/β-Catenin Pathway inhibitor, XAV939, assessed the DPCs proliferation by CCK-8 assay. And the cell cycle was also analyzed by flow cytometry. We found that Wnt10b could promote elongation of the hair shaft, whereas XAV-939 treatment could eliminated this phenomenon. AdWnt10b treatment promoted the proliferation and induced G1/S transition of DPCs. AdWnt10b stimulation up-regulated β-Catenin protein in DPCs. Inhibition of Wnt/β-Catenin signaling by XAV-939 could decreased the basal and Wnt10b-enhanced proliferation of DPCs. And could also suppress the cell cycle progression in DPCs. In summary, our study demonstrates that Wnt10b could promote HFs growth and proliferation of DPCs via the Wnt/β-Catenin signaling pathway in Rex rabbits.

## Introduction

Hair follicles (HFs) contain dermal papilla cells (DPCs) and epithelial cells, which are the two major types of cell [1]. During them, DPCs regulate HF development and growth, instruct the matrix cells to proliferate, move upward and differentiate into the outgrowing hair shaft and the inner root sheath [2]. DPCs from the postnatal skin retain the ability to direct epithelial cells [3]. Moreover, formation of new DP can be induced in adult skin by activating the Wnt pathway in the epidermis [4].

Hair development and subsequent cycling follow a carefully choreographed rhythm with cycling controlled by multiple genes and pathways [5]. Several families of secreted signaling molecules have been implicated in the communication between the epidermis and dermis during HF development [6]. Among these processes, Wnt/β-Catenin signaling has been shown to be required for the initiation and regeneration of HF development in mice [7]. In the Wnt/β-Catenin signaling pathway, β-Catenin takes over an important role in the formtion of HFs. When β-Catenin accumulates in the cytoplasm, it can be translocated to the nucleus, where it interacts with the TCF/LEF transcription factors to activate gene expression [8]. However, the formation of placodes that generate HF is blocked if the β-Catenin is mutated during embryogenesis [9].

In HF development, the Wnt family appears to be the earliest and the most critical regulator for early development of the epidermis [10]. Wnt proteins are expressed in the embryonic skin of mice [11]. Wnt10b was initially expressed uniformly in the epidermis and is markedly up-regulated in follicular placodes [12].

In mice, Wnt10b has been shown to promote differentiation of primary skin epithelial cells toward the hair shaft and inner root sheath of the HF cells *in vitro* [13,14], and also to promote HFs growth via canonical Wnt signalling pathway [15]. To understand the role of Wnt10b in the HFs of Rex rabbits, the present study investigated the effects of inhibition and activation of the Wnt/β-Catenin signaling pathway in HF shafts growth and the DPCs proliferation from Rex rabbits.

## Materials and methods

### Ethical approval of the study protocol

All experiment including animal experiments were carried out at Shandong Agricultural University. The ethics approval was also obtained from Shandong Agricultural University (Shandong, China, Approved number: SDAUA-2017-029) and were carried out in accordance with the Guidelines for Experimental Animals of the Ministry of Science and Technology (Beijing, China). All surgical procedures were carried out according to recommendations proposed by the European Commission (1997), and all efforts were made to minimize the suffering of animals.

### Rabbit anesthesia and sedation

Four-week-old Rex rabbit was anesthetized by intravenous injection of diazepam (1.6 mg/kg) and pentobarbital sodium (30 mg/kg) followed by cervical dislocation. The anesthetic effect was assessed by the indicators include smooth breathing, muscle relaxation, no pain response, and miosis. Rabbit was confirmed dead when no breathing or heart beat was detected. And then the skin samples were taken away from the whisker or dorsal back position of the rabbit immediately for the next treatment.

### Organ culture of HFs

We used whisker HFs for the organ cultures. Whisker HFs of a 4-week-old Rex rabbit was isolated as has been previously described for mice [16]. Early or mid-anagen growth phase follicles were selected for culture, and the part of the hair shaft that extended over the epidermal surface was cut off. HFs were plated in 24-well plates with one follicle per well at 31°C saturated humidity air temperature with 5% CO_2_ and 95% box in the general culture and cultured in one of the following basal media: Williams E medium (GIBCO, U.S.A.), penicillin–streptomycin (Solarbio, China), insulin (GIBCO, U.S.A.), hydrocortisone (Sigma, Germany), and L-glutamine (GIBCO, U.S.A.); basal medium containing AdWnt10b (Hanbio Co. LTD, China) and AdGFP (Hanbio Co. LTD, China) at ultimate titers of 10^8^; or basal medium containing XAV-939 (10 µmol/l)(Sigma, Germany) and AdWnt10b plus XAV-939. After 2 days, every culture was replaced with fresh media, and the length of the outgrowing hair shafts was determined by analyzing the digital images at 5 days of growth with stereo microscope (Nikon SMZ800N, Japan).

### Isolation and culture of DPCs

Small skin pieces were obtained from 4-week-old rabbits. And then the DPCs were isolated based on the previously reported method [17] and cultured in 6-well plates. The isolated DPCs were cultured in the basal medium of DMEM (Gibco, C11995500BT, U.S.A.) containg 10% fetal bovine serum (FBS) (SeraPro, S601s, U.S.A.) and 1% Penicillin–Streptomycin (Solarbio, #P1400, China) at 37°C saturated humidity air temperature with 5%CO2 and 95% box in the general culture.

### Giemsa staining

The third generation of DPCs was plated on cover glass (WHB, China) in 6-well plates for culturing 3 days. Removed the basal medium and rinsed for three times using phosphate buffer solution (PBS). Added 2–3 drops of Wright-Giemsa Stain solution (Solarbio, #G1020, China) for rinsing 2 min. And then washed with water, dried with the hydro-paper and observed the cell morphology by using a fluorescence microscope (Nikon ECLIPSE 80i, Japan).

### Immunofluorescence staining

The third generation of DPCs was plated on cover glass (WHB, China) in 6-well plates for culturing 4 days. Removed the basal medium and rinsed for three times using phosphate buffer solution (PBS). And then fixed with 4% paraformaldehyde (Solarbio, #P1110, China) for 30 min in room temperature and washed with PBS for three times, permeabilized with 0.5% Trixton X-100 for 15 min in room temperature and washed with PBS for three times, blocked with goat serum (BOSTER, #12C09A, China) for 30 min and incubated with α-SMA antibody (BOSTER, #BM0002, China) /or Vimentin antibody (BOSTER, #BM0135, China) /or Wnt10b antibody (orb97574, biorbyt, U.S.A.) overnight at 4°C in wet box [18–20]. For immunofluorescence staining, we used SABC-FITC SP kit (BOSTER, #SA1062, China). The DPCs were incubated with goat anti mouse IgG secondary antibody for 30 min at 37°C and then added SABC-FITC for incubating at 37°C in darkness for 30 min after washing with PBS. Counter-staining with DAPI and mounting with anti-fluorescence quenching agent (Beyotime, #P0128, China). The fluorescence signals were observed by using a fluorescence microscope (Nikon ECLIPSE 80i, Japan).

### Treatment of DPCs

For overexpression of Wnt10b treatment, the DPCs were cultured with AdWnt10b at the concentration of 300 multiplicity of infection (MOI) for 2 h and then refresh the new basal medium. For inhibition of Wnt/β-Catenin pathway, XAV-939 (MCE, #HY-15147, U.S.A.) at a concentration of 10 μM was added into the basal medium 4 h after transfection.

### Proliferation of DPCs/CCK-8

DPCs were plated in a 96-well plate at a density of 4000 cells/well and cultured in basal medium for 24 h, and then followed by the indicated treatment. Ten microliters Cell Counting Kit-8 (CCK-8) was added to each well at 24 h after treatment, and incubated the plate for 4 h at 37°C. The optical density at 450 nm was read by an microplate reader (BioTek Elx-808, U.S.A.).

### Cell cycle analysis/flow cytometry

For flow cytometry analysis, the DPCs were treatment followed by the indicated treatment for 4 days. The cells were digestion by trypsin and fixed in 70% cold ethanol for at least 18 h, centrifuged at 500 ***g*** for 5 min, dropped the supernate and added 0.5 ml propidium iodide (PI) (Cat: 550825, BD) for incubating 15 min at 37°C in dark. The cells were then analyzed by flow cytometry (BD Accuri C6, BD). And the results were analyzed with the ModFit LT 5.0 software.

### Western blotting analysis

Total protein of DPCs was extracted with the cell active protein extraction kit (Sangon Biotech, #C500022, China). And the protein concentration was measured with a BCA Protein Assay Kit (Beyotime, China). For the Western blot analysis, equal protein amounts (40 µg per well) were denatured for 10 min at 100°C and separated with 12% gel electrophoresis. The protein was transferred onto a polyvinylidene fluoride (PVDF) microporous membrane at 200 mA and 4°C for 2 h. After 1 h of blocking in block solution (Beyotime, China) at room temperature, membranes were incubated at 4°C overnight in the following primary antibodies: Wnt10b antibody (orb97574, biorbyt, U.S.A.); β-catenin antibody (Cat. #06-734, Millipore, U.S.A.); Tubulin antibody (Beyotime, #AT819, China). The blots were then soaked with anti-mouse IgG-conjugated horseradish peroxidase (Beyotime, China) for 4 h at 4°C, and the protein bands were detected by Super Signal West Femto Maximum Sensitivity Substrate (Thermoscientific) or ECL (Beyotime, China). The results were visualized by exposing the blots to X-RAY film (Kodak). The films were scanned by a scanner (HP ScanJet 6100C) and signal intensity (Quantification) was conducted using ImageJ 1.43 software (National Institutes of Health, Bethesda, MD).

### Statistical analysis

All the data were analyzed with SAS software (SAS version 8e; SAS Institute, Cary. NC, U.S.A.). A one-way ANOVA model was used to evaluate the means among various groups. The data were shown as means ± SEM. *P* < 0.05 was regarded as statistically significant.

## Results

### Wnt10b promotes HF growth *in vitro*

**Figure 1:**
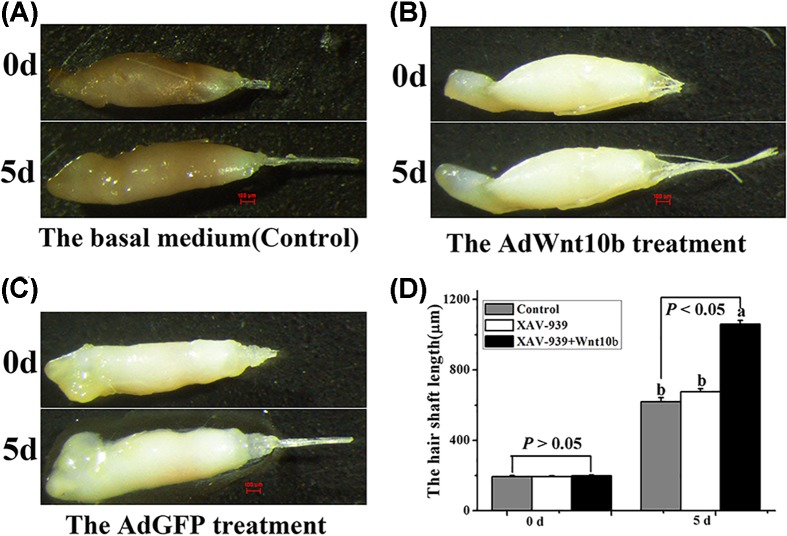
The effects of AdWnt10b on vibrissal follicles Morphology of HFs with different treatments, including (**A**) The basal medium (control); (**B**) The AdWnt10b treatment. (**C**) The AdGFP treatment; (20× magnification). (**D**) Analysis of hair shaft growth with different treatments; (*n* = 12); d: days.

Isolated Rex rabbit whisker HFs at early or mid-anagen were cultured for 5 days with AdWnt10b, AdGFP or the basal medium. After 5 days, we examined the shaft growth of the cultured HFs (Figure 1A–C). We found that whisker HFs co-cultured with AdWnt10b grew faster than follicles cultured in basal medium or co-cultured with AdGFP (*P* < 0.0001) (Figure 1D).

### Overexpressed Wnt10b promotes proliferation of DPCs

**Figure 2:**
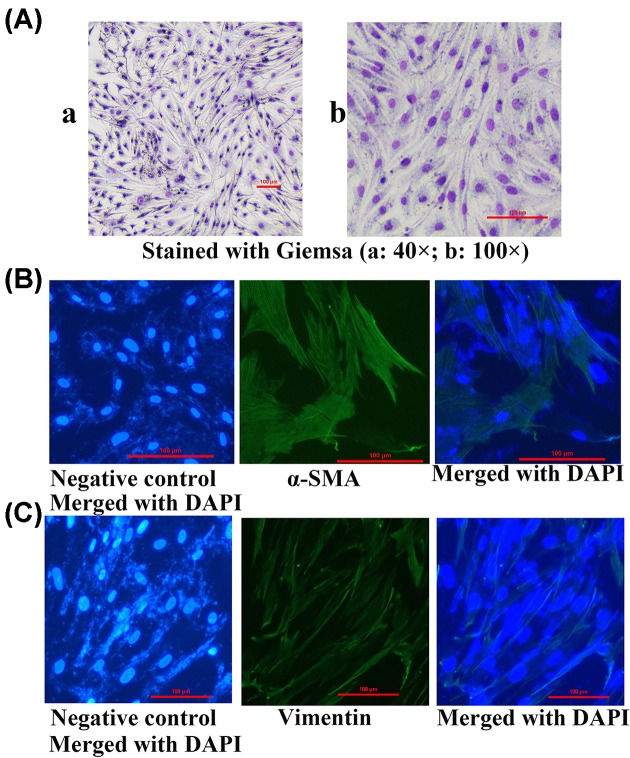
Characterization of DPCs DPCs were isolated form 4-week-old Rex rabbit and cultured *in vitro*. The third generation of DPCs were characterized by Giemsa staining to observe the cell metamorphosis (**A**), and by immunofluorescence staining for the expression of α-SMA (100× magnification) (**B**), by immunofluorescence staining for the expression of Vimmentin (100× magnification) (**C**).

**Figure 3:**
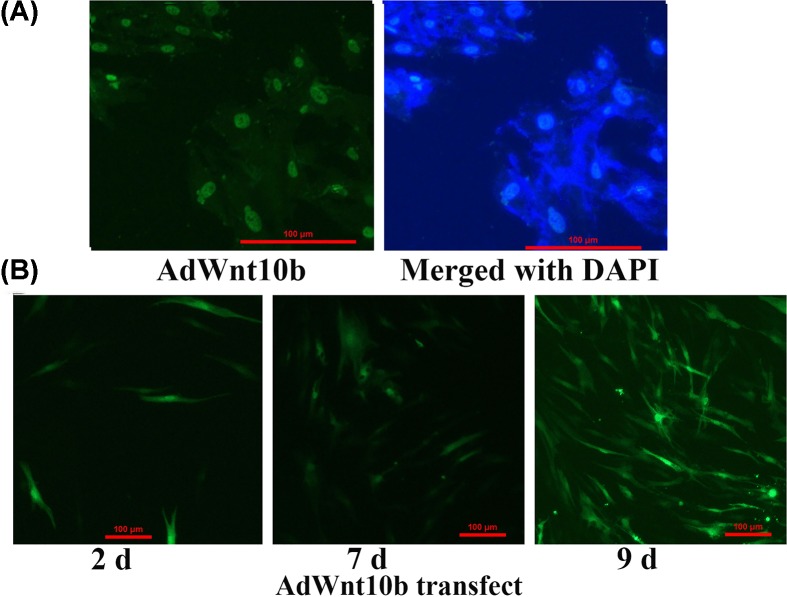
The effects of Wnt10b protein expression and AdWnt10b transfection on DPCs (**A**) Immunofluorescence localization of Wnt10b expression in DPs. The third generation of DPCs were cultured *in vitro*, and the Wnt10b protein expression was localized by immunofluorescence staining in DPCs (100× magnification). (**B**) The transfection effect of AdWnt10b on DPCs. The medium was refresh with the basal media after transfection with AdWnt10b for 2 h in DPCs. And the GFP was observed by inversion fluorescence microscope after 2 days, 7 days and 9 days (40× magnification). d: days.

**Figure 4:**
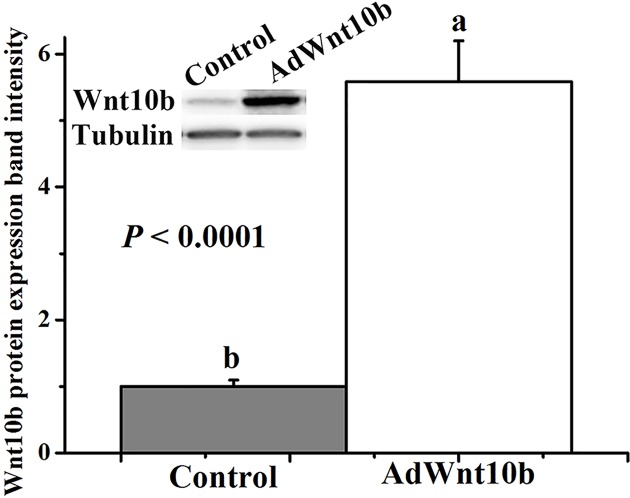
The expression band intensity of Wnt10b protein in DPCs The Wnt10b protein expression between endogenous (Control) and overexpressed (AdWnt10b) in DPCs was also tested at 9 days after transfected with the AdWnt10b for 2 h. Data are the means ± SEM (stand error of mean). a, b Means with different superscripts differ (*P* < 0.05).

**Figure 5:**
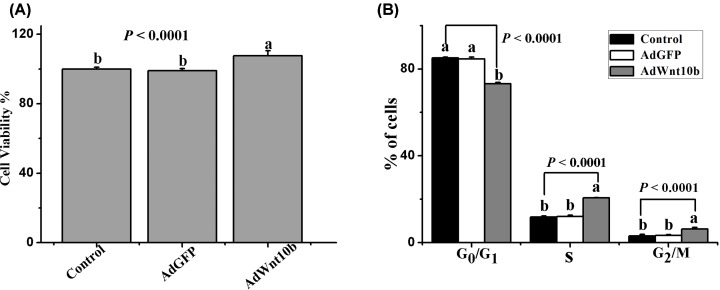
Wnt10b promotes DPCs proliferation and induces the G1/S transition (**A**) DPCs were treated with AdWnt10b, and the proliferation of DPCs were measured by the CCK-8 assay. (**B**) The cell cycle was also analyzed by flow cytometry. Data are the means ± SEM (stand error of mean). a, b, c Means with different superscripts differ (*P* < 0.05).

To investigate the effects of Wnt10b on HFs growth, DPCs were isolated form 4-week-old rabbits and were identified by Giemsa staining (Figure 2A), α-SMA (Figure 2B) and Vimentin (Figure 2C). The Wnt10b was also assessed by immunofluorescent staining before and after transfection of adenovirus in DPCs (Figure 3A,B). And the Wnt10b protein expression between endogenous and overexpressed in DPCs was also tested at 9 days after transfected with the AdWnt10b for 2 h. The result showed that the AdWnt10b transfection significantly upregulated the Wnt10b protein expression in DPCs (*P* < 0.0001) (Figure 4). DPCs were cultured followed by the indicated treatment and the cell proliferation was assessed by the CCK-8. As shown in Figure 5A, AdWnt10b treatment significantly enhanced the cell proliferation (*P* < 0.0001). Cell cycle analyzed by flow cytometry revealed that AdWnt10b treatment could reduced the number of cells in G_0_/G_1_ phase (*P* < 0.0001) and promoted the cells into S and G_2_/M phase (*P* < 0.0001) (Figure 5B).

### Wnt10b activates the Wnt/β-catenin pathway

**Figure 6:**
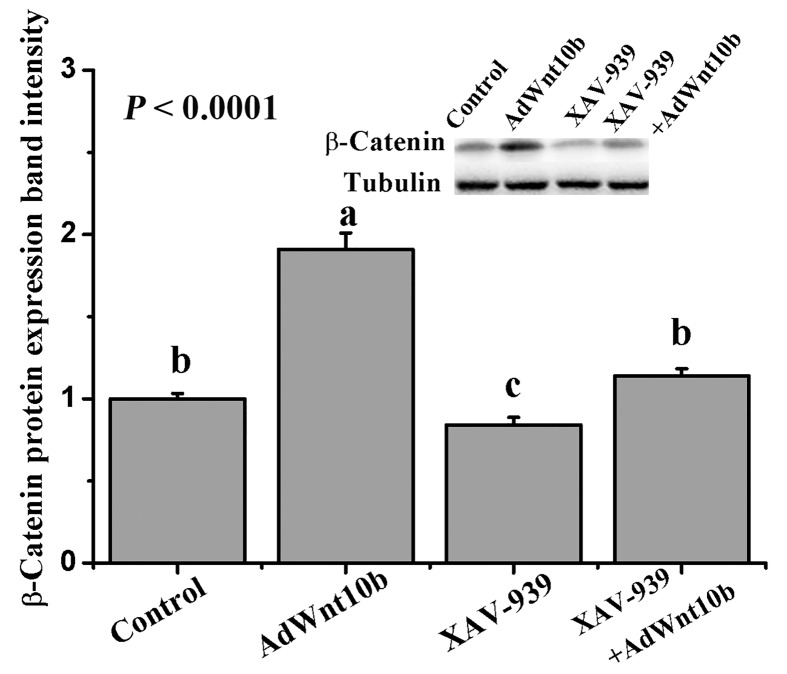
Wnt10b activates the Wnt/β-catenin pathway The protein expression of β-catenin in DPCs with AdWnt10b and/or XAV-939 treatment was detected by Western blotting. Data are the means ± SEM (stand error of mean). a, b, c Means with different superscripts differ (*P* < 0.05).

As shown in Figure 6, AdWnt10b transfection up-regulated the protein expression of β-catenin in the DPCs (*P* < 0.0001). Treatment with XAV-939, which is a specific inhibitor of Wnt/β-catenin signaling that promotes the degradation of β-catenin to block the Wnt/β-catenin signaling pathway [21], could reduce the β-catenin protein expression in DPCs (*P* < 0.0001).

### Wnt10b promotes HF growth and DPCs prolifreration via Wnt/β-catenin pathway

**Figure 7:**
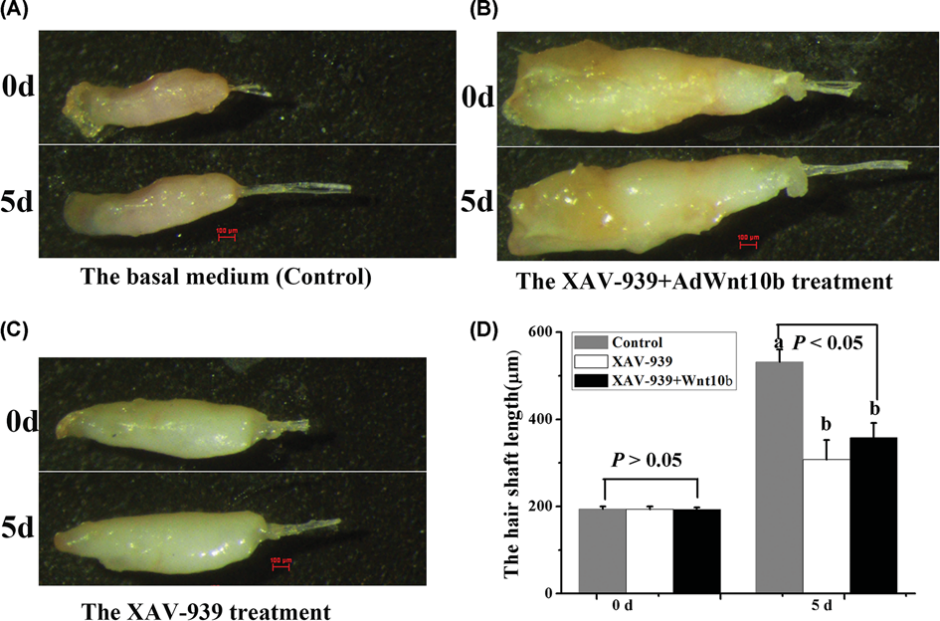
The effects of AdWnt10b and/or XAV-939 on vibrissal follicles Morphology of HFs with different treatments. (**A**) The basal medium (control). (**B**) The XAV-939+Wnt10b treatment. (**C**) The XAV-939 treatment; (20× magnification). (**D**) The analysis of hair shaft growth with different treatments; (*n* = 12); d: days.

To determine which signaling pathway Wnt10b uses to promote HF growth in Rex rabbits, we used XAV-939, which is a specific inhibitor of Wnt/β-catenin signaling. When we cultured whisker HFs with XAV-939 or AdWnt10b plus XAV-939, we found that the whisker HFs treated with XAV-939 or AdWnt10b plus XAV-939 grew slower than those cultured in basal medium (*P* < 0.0001) (Figure 7A–D).

**Figure 8:**
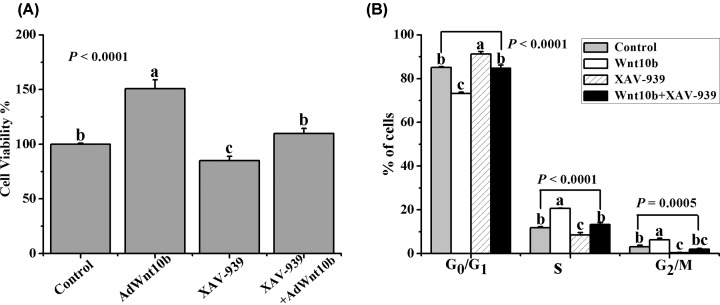
Wnt10b promotes DPCs prolifreration via Wnt/β-catenin pathway (**A**) DPCs were treated with XAV-939 and/or AdWnt10b, and the proliferation of DPCs were measured by the CCK-8 assay. (**B**) The cell cycle was also analyzed by flow cytometry. Data are the means ± SEM (stand error of mean). a, b, c Means with different superscripts differ (*P* < 0.05).

To further investigated the role of Wnt/β-catenin signaling pathway in Wnt10b promoting proliferation of DPCs, the DPCs were treatment with XAV-939 and the cells proliferation was assessed by CCK-8. As shown in (Figure 8A), inhibition of Wnt/β-catenin by XAV-939 significantly decreased the DPCs proliferation rate (*P* < 0.0001). Cell cycle analyzed by flow cytometry also revealed that the promotion of DPCs proliferation by Wnt10b was inhibited by XAV-939 (*P* < 0.0001) (Figure 8B).

## Discussion

HFs are known to be constantly renewed with alterations between the phases of anagen, catagen, and telogen [16]. In HFs, the balance between activators and inhibitors may determine whether a HF can re-enter anagen [22]. Wnts are expressed in HFs throughout life from embryo to adulthood, and they are considered to be critical for HF development and maturation [12,23]. In mice, testing the effects of Wnts (Wnt3a, 5a, 10b, 11) showed that only Wnt10b demonstrated obvious promotion of epithelial cell differentiation and hair shaft growth [24]. Additionally, Wnt10b also had a potent ability to sustain the expression of versican in DPCs and maintained their HF induction ability [25].

In our study, we examined the effects of Wnt10b on developed HFs in Rex rabbits. For this purpose, we used organ cultures of whisker HFs because they have been shown to be cultured for a prolonged period [21]. Like what was observed in mice HFs [16], AdWnt10b transfection could promote the elongation of the hair shaft in organ cultures. The DPC, which not only regulates HF development and growth, but is also considered as a reservoir of pluripotent stem cells [2], is active and progenetive during anagen phase [17]. In the present study, the DPCs grew faster in response to Wnt10b. These results suggested that Wnt10b may promote HF growth through induce the DPCs proliferation.

Wnt signals have been implicated in the control of HF development. And the Wnt/β-catenin signals are generally required for the initiation, development, and generation of HFs [7]. β-Catenin, which has been implicated in skin and HF development, is an essential molecule in Wnt signal. And the stem cells also require β-catenin in order to differentiate into HFs [9]. Wnt/β-catenin signal is mediated by the co-activity of transcription factors β-catenin and LEF1. If β-catenin is absence in the developing mouse epidermis, the HF formation is inhibited [26]. In our study, XAV-939 was used to block the Wnt/β-catenin signals through promoteing the degradation of β-catenin to prevent forming the β-catenin/LEF1/TCF. We found that Wnt10b could significantly promote the elongation of the hair shafts and the differentiation of DPCs. While inhibition of Wnt/β-Catenin signaling by XAV-939 could eliminate this enhanced promotion phenomenon. WB results also showed that Wnt10b induced the β-catenin protein expression in DPCs. Morever, the up-regulation of β-catenin induced by Wnt10b was inhibited by XAV-939. These results suggest that Wnt10b is likely to be involved in Wnt/β-catenin signaling pathway in the DPCs of Rex rabbits. And it also implies that β-catenin is an essential signal for the proliferation of DPCs.

## Conclusion

In conclusion, Wnt10b can promote growth of Rex rebbits HFs *in vitro* and enhance the proliferation of DPCs *in vitro* via the Wnt/β-catenin signaling pathway. We believed that Wnt10b was deeply involved in the Rex rabbit HF development through the Wnt/β-Catenin signaling pathway.

## Abbreviations

CCK-8: Cell Counting Kit-8
DPC: dermal papilla cell
FBS: fetal bovine serum
HF: hair follicle
MOI: multiplicity of infection
PBS: phosphate buffer solution
PI: propidium iodide
PVDF: polyvinylidene fluoride
